# Longitudinal associations between childhood maltreatment and suicidal ideation in youth: the roles of complex posttraumatic stress disorder symptoms and interpersonal factors

**DOI:** 10.3389/fpsyt.2026.1976531

**Published:** 2026-10-14

**Authors:** Qingyun Yu, Yang Li, Li Wang, Juncheng Guo, Wenchao Wang

**Affiliations:** 1 Normal College, Jingchu University of Technology, Jingmen, China; 2 Research Team of Integrated Mental Health Education for Primary, Secondary, and Higher Education, Jingchu University of Technology, Jingmen, China; 3 Beijing Key Laboratory of Applied Experimental Psychology, Faculty of Psychology, Beijing Normal University, Beijing, China

**Keywords:** childhood maltreatment, suicide, CPTSD, interpersonal needs, youth

## Abstract

**Background:**

Globally, suicide ranks as the third leading cause of death among youth, with risk particularly pronounced among those who have experienced childhood maltreatment. Guided by the developmental model of suicide risk and the interpersonal theory of suicide, this study aimed to examine the link between childhood maltreatment and suicidal ideation in youth, and to investigate the mediating roles of complex posttraumatic stress disorder (CPTSD) symptoms and interpersonal needs.

**Methods:**

Three-wave longitudinal data from 1,427 college students (53.7% female) with childhood maltreatment were included. Participants completed self-report measures assessing childhood maltreatment, CPTSD symptoms, perceived burdensomeness (PB) and thwarted belongingness (TB), and suicidal ideation. Structural equation modeling was conducted to evaluate the proposed pathways.

**Results:**

Findings indicated that PB, rather than TB, functions as the primary proximal risk factor for suicidal ideation. Furthermore, within CPTSD, disturbances in self-organization (DSO), rather than posttraumatic stress (PTS), mediated the association between childhood maltreatment and PB. Specifically, childhood maltreatment was indirectly associated with PB through the negative self-concept dimension of DSO, and PB was subsequently positively associated with suicidal ideation.

**Conclusions:**

These findings suggest that, for youth with childhood maltreatment, DSO may be even more critical than PTS in understanding suicidal ideation. Intervention strategies targeting negative self-concept may be particularly effective in reducing suicidal ideation among youth.

## 1 Introduction

Youth suicide remains a critical public health problem and is currently the third leading contributor to mortality among this population (1). Among youth who have experienced childhood maltreatment, the prevalence of suicidal ideation is 2.36 times higher, and the rate of suicide attempts is 2.91 times higher, compared to those non-maltreated (2). Childhood maltreatment, referring to abuse and neglect perpetrated by parents or caregivers, is widely recognized as a major predictor of adverse psychological outcomes (3). Research indicates that more than 60% of emerging adults, individuals in the critical developmental transition from adolescence to adulthood, have experienced childhood maltreatment (4). The developmental model of suicide risk further proposes that the pathway through which childhood maltreatment confers suicide risk among youth is highly complex (5). Accordingly, it is essential to clarify the processes that contribute to suicide vulnerability among emerging adults who have experienced childhood maltreatment in order to guide the development of targeted prevention and intervention efforts.

The developmental model of suicide risk proposes that internalizing symptoms play a key intermediary role in the association between childhood maltreatment and suicidal ideation (5). As a prototypical, chronic, and deleterious traumatic experience, childhood maltreatment invariably contributes to a range of posttraumatic internalizing psychopathology (6, 7). In recent years, more and more scholars have focused on the role of complex posttraumatic stress disorder (CPTSD) symptoms (8, 9). CPTSD symptoms emerge in response to chronic, repetitive, and interpersonal traumatic experiences and encompass both posttraumatic stress symptoms (PTS; including a persistent sense of current threat, avoidance of trauma-related cues, and reexperiencing of past traumas) and disturbances in self-organization (DSO; including relational disruptions, negative self-concept, and affective dysregulation) (10). Research has shown that among survivors of childhood maltreatment, the prevalence of CPTSD is three to four times higher than among individuals diagnosed with PTSD alone (11). This emphasizes the value of further research on CPTSD among childhood maltreatment survivors.

Complex posttraumatic stress disorder symptoms are also strongly associated with suicidality. One study examining the relationship between sexual abuse and suicide risk found that CPTSD played a significant mediating role (12). Additional research has demonstrated that CPTSD mediates the association between trauma exposure and suicide risk (13). Moreover, findings from a multinational study indicate that, among childhood maltreatment survivors, CPTSD exerts a substantially stronger influence on suicide risk than PTSD symptoms alone, indicating a more prominent role for DSO (14). However, some existing studies treat CPTSD as a unitary construct (15, 16), thereby overlooking the potentially distinct contributions of PTS and DSO. For instance, chronic and repetitive interpersonal trauma is more strongly associated with the cooccurrence of both PTS and DSO than with PTS alone (17). Although some studies have differentiated the developmental pathways of PTS and DSO in linking childhood experiences to suicidal ideation (18), PTS and DSO can further be disaggregated into finer-grained subdimensions, which may exhibit differential associations with suicidality (19). Therefore, more granular research is needed to precisely delineate the roles of specific symptom dimensions and identify more effective targets for intervention.

However, as a mental disorder or symptom, CPTSD likely represents a distal rather than proximal risk factor for suicide (20, 21); thus, proximal mechanisms warrant further consideration. The Interpersonal Theory of Suicide posits two proximal factors that may further elucidate this relationship (22). These are thwarted belongingness (TB, the absence of mutually supportive bonds alongside a perceived state of being socially isolated) and perceived burdensomeness (PB, the perception of being a burden to others) (22). Elevated levels of both TB and PB jointly increase the likelihood of developing suicidal ideation. The interpersonal theory has been robustly validated among emerging adults (23, 24). However, no existing studies have tested the potential link between CPTSD and the constructs of the interpersonal theory. Nevertheless, insights may be drawn from existing evidence on PTSD. Numerous studies have demonstrated that elevated PTSD symptoms are associated with greater TB and PB, which in turn exacerbate suicidal ideation (25, 26). However, some research suggests that specific PTSD symptom clusters may exhibit differential associations with TB and PB. For example, avoidance and hyperarousal symptoms are more likely to lead individuals to withdraw from social interactions or remain hypervigilant toward others, thereby impairing interpersonal functioning (27). Extending this logic to CPTSD, PTS and DSO dimensions may exert distinct influences on these interpersonal constructs. However, this distinction has not yet been empirically examined.

In summary, current understanding of the pathways linking childhood maltreatment to suicidal ideation among youth remains limited, and no study to date has examined the potential sequential associations of CPTSD and interpersonal needs with suicidal ideation in this population. Drawing on the Developmental Model of Suicide Risk and integrating the Interpersonal Theory of Suicide, the present study was guided by an *a priori* theoretical expectation that childhood maltreatment would be associated with suicidal ideation through CPTSD symptoms and interpersonal needs. Accordingly, our primary research question was whether the hypothesized sequential pathway from childhood maltreatment through CPTSD and interpersonal needs to suicidal ideation could be observed among emerging adults.

Beyond this theory-driven primary model, we further sought to disentangle the potential contributions of the two core dimensions of CPTSD—PTS and DSO—and their respective subdimensions. Given the limited empirical evidence regarding which specific CPTSD dimensions may be involved in the association between childhood maltreatment and suicidal ideation, these component-level analyses were considered exploratory. Thus, we did not specify directional hypotheses regarding the relative contributions of PTS, DSO, or their individual subdimensions.

## 2 Methods

### 2.1 Participants and procedure

The present sample was recruited as part of a study on college students’ mental health. Data were collected from a central China university that enrolls students from all regions of the country and represents a mid-level educational institution nationally, enhancing the generalizability of the findings. A total of 4,185 freshmen completed W1, of whom 2,669 met the prespecified cutoff for at least one subtype of childhood maltreatment and were subsequently included in the longitudinal follow-up and analyses. Given the trauma-specific focus of CPTSD, the present study specifically examined participants with qualifying childhood maltreatment histories. Prior to data collection, an oral explanation of the study procedures and voluntary participation was provided. Students who did not wish to participate were free to leave the classroom. Those who remained completed an anonymous online survey using a mobile application, with trained psychology teachers overseeing the process. The survey required approximately 20 minutes to finish. Participants younger than 18 years provided informed consent together with their legal guardians, whereas participants aged 18 years or older provided informed consent themselves, and authorization for the current investigation was formally obtained from the university’s ethics committee. In addition, students were informed that school-based psychological or counseling support could be accessed if necessary. At Wave 1 (W1), participants provided demographic information, past negative life events, childhood maltreatment, CPTSD. Interpersonal needs were assessed at Wave 2 (W2), whereas suicidal ideation was measured at Wave 3 (W3) during the follow-up period. Each adjacent wave was separated by approximately six months.

A total of 4,185 freshmen completed the W1 survey. According to the cutoff thresholds proposed by Tietjen et al. (28), 2,669 individuals (63.7% of the sample) met the criteria for at least one subtype of childhood maltreatment on the Childhood Trauma Questionnaire-Short Form (sexual abuse ≥ 6, emotional neglect ≥ 10, emotional abuse ≥ 9, physical abuse ≥ 8, physical neglect ≥ 8). However, due to class/major adjustment and school suspensions, only 2366 (88.6%) and 1730 (64.8%) participated in the W2 and W3 survey. Results from Little’s (29) missing completely at random (MCAR) test suggested that the distribution of missing data did not deviate significantly from a random pattern, χ^2^ (17) = 26.48, *p* = .066, χ^2^/df = 1.56 < 2. Following this, independent-samples *t* tests were conducted to further examine whether participants with and without missing data differed on the study variables. Significant differences were observed for baseline age, *t*(2667) = 4.12, *p* <.001, and baseline childhood maltreatment, *t*(2667) = 2.07, *p* = .039. No significant differences were found for baseline past negative life events, *t*(2667) = -0.37, *p* = .708, or baseline family monthly income, *t*(2667) = 1.75, *p* = .080. However, all effect sizes were small (all < 0.25), indicating limited practical differences. Accordingly, analyses were conducted on the 1,427 participants who completed assessments across all three waves. Their demographic characteristics are shown in **Table 1**.

**Table 1:** Demographic characteristics of the sample.

Characteristics	*n*	%
Sex		
Male	660	46.3
Female	767	53.7
Age		
18 and below	771	54.0
19	355	24.9
20	76	5.3
21	127	8.9
22 and above	98	6.9
Resident		
Urban	341	23.9
Rural	1,086	76.1
Family monthly income		
Less than 1000 RMB	58	4.1
1,000-3,000 RMB	306	21.4
3,000-6,000 RMB	504	35.3
6,000-10,000 RMB	358	25.1
10,000-15,000 RMB	128	9.0
15,000-20,000 RMB	36	2.5
More than 20,000 RMB	37	2.6

### 2.2 Measures

#### 2.2.1 Childhood maltreatment

Childhood maltreatment prior to college was evaluated using the Childhood Trauma Questionnaire-Short Form developed by Bernstein et al. (30). Each item is rated on a 5-point Likert scale (1 = *never* to 5 = *very often*), with higher summed scores reflecting greater exposure to maltreatment. In the present sample, the measure showed acceptable internal consistency (Cronbach’s α = .78).

#### 2.2.2 CPTSD symptoms

In this study, the International Trauma Questionnaire was used to measure the CPTSD of participants (31). This questionnaire has been shown to have good reliability and construct validity among Chinese populations (32). The scale consists of two subscales. It is divided into PTS (six items) and DSO (six items). These items are measured on a 5-point Likert scale (0 = not at all to 4 = extremely). Higher total scores of the PTS and DSO subscales indicate more severe symptoms. The internal consistencies were good for PTS (Cronbach’s α = .91) and DSO (Cronbach’s α = .91).

#### 2.2.3 PB and TB

The study employed the Interpersonal Needs Questionnaire to evaluate two key interpersonal constructs: perceived burdensomeness and thwarted belongingness (33). This questionnaire has been shown to have good reliability and construct validity among Chinese populations (34). The scale consists of two subscales. It is divided into PB (six items) and TB (nine items). Items are rated on a 7-point Likert scale (1 = completely disagree to 7 = completely agree). Higher total scores of the subscale indicate a higher level of PB or TB. In this study, the internal consistencies were good for PB (Cronbach’s α = .95) and TB (Cronbach’s α = .84).

#### 2.2.4 Suicidal ideation

Suicidal ideation was measured using the four-item subscale of the Hopeless Depression Questionnaire developed by Metalsky and Joiner (35). This questionnaire has been shown to have good reliability and construct validity among Chinese populations (36). Each item is rated on a 4-point scale ranging from 0 (e.g., I do not have thoughts of killing myself) to 3 (e.g., I always have thoughts of killing myself). Higher summed scores reflect greater severity of suicidal ideation. In this study, the internal consistencies were good for suicidal ideation (Cronbach’s α = .92).

#### 2.2.5 Covariables

Demographic characteristics, including sex, age, residence, and family income, have been reported to be associated with CPTSD symptom severity and suicidal ideation in earlier studies (18, 37). Therefore, these variables were included as covariates in the model. In addition, participants’ past negative life events were also controlled for, as measured by the Negative Life Events Scale (38). The 25-item scale assesses a series of adverse life events that may have occurred before age 18 among Chinese emerging adults (e.g., “Being punished by teachers”), rated on a 6-point scale (0 = never happened to me to 5 = extremely). One item related to childhood abuse and neglect was removed to avoid conceptual overlap with the main study variables. The scale demonstrated excellent internal consistency in the current study (Cronbach’s α = .96).

### 2.3 Data analysis

Data analyses were performed using R (Version 4.0.3) and Amos 24.0. The final sample contained no missing values, since incomplete questionnaires were filtered out by the survey system. Initially, descriptive and correlational analyses were applied to assess relationships among variables.

Structural equation modeling (SEM) was conducted to examine whether PTS/DSO and PB/TB mediated the relationship between childhood maltreatment and suicidal ideation. SEM was chosen because it allows theory-driven models to be evaluated. In addition, to examine the specific contributions of different PTS/DSO dimensions, each disorder was further divided into three components according to ICD-11 criteria. Finally, to assess the indirect effects, we performed a 5,000-resample bias-corrected bootstrap analysis in line with the procedure proposed by Hayes and Preacher (39).

## 3 Results

### 3.1 Preliminary analysis

An overview of descriptive statistics and correlations is provided in **Table 2**. Childhood maltreatment, PTS, DSO, TB, PB, and suicidal ideation were all significantly correlated with each other, except for the association between TB and suicidal ideation.

**Table 2:** The means, standard deviations and intercorrelations among variables.

Variables	*M*	*SD*	1	2	3	4	5	6
1. W1 CM	39.8	9.08	1					
2. W1 PTS	9.26	4.68	.17^***^	1				
3. W1 DSO	10.7	5.29	.19^***^	.56^***^	1			
4. W2 TB	31.6	10.6	.24^***^	.06^*^	.08^**^	1		
5. W2 PB	9.92	5.97	.15^***^	.19^***^	.33^***^	.15^***^	1	
6. W3 SI	0.22	0.96	.07^**^	.12^***^	.13^***^	0.04	.17^***^	1
7. ^a^Sex	–	–	-.12^**^	-.02	.01	-.22^**^	-.05	-.03
8. ^b^Age	–	–	.06^*^	-.08^**^	-.15^***^	.06^*^	-.06^*^	-.05^*^
9. ^c^Residence	–	–	0.03	-0.02	-0.01	-0.01	0.02	-0.02
10. ^d^Family income	3.31	1.24	-0.05	-0.03	-.06^*^	-0.02	-0.04	-0.04
11. Past negative events	39.7	20.5	.27^***^	.23^***^	.24^***^	.07^**^	.13^***^	.10^**^

M, Mean, SD, Standard deviation, W1, Wave 1, W2, Wave 2, W3, Wave 3, CM, Childhood maltreatment, PTS, Posttraumatic stress, DSO, Disturbances in self-organization, TB, Thwarted belongingness, PB, Perceived burdensomeness, SI, Suicidal ideation. ^a^1 = Male, 2 = Female. ^b^1 = 18 years and younger, 2 = 19 years, 3 = 20 years, 4 = 21 years, 5 = 22 years and older. ^c^1 = City, 2 = Rural area. ^d^1 = 1000 CNY and under, 2 = 1000-3000 CNY, 3 = 3000-6000 CNY, 4 = 6000-10000 CNY, 5 = 10000-15000 CNY, 6 = 15000-20000 CNY, 7 = 20000 CNY and above. ^*^p < .05, ^**^p < .01, and ^***^p < .001.

### 3.2 Structural equation modeling analysis

First, we examined the mediating role of PTS/DSO and TB/PB between childhood maltreatment and suicidal ideation (see **Figure 1**). The model fit was acceptable: χ^2^/*df* = 1.767, CFI = .998, TLI = .968, RMSEA [90% CI] = .023 [.000,.055]. The results showed that childhood maltreatment is significantly associated with PTS, DSO, TB, and PB. DSO is significantly associated with PB and PB is significantly associated with suicidal ideation. The indirect effects of the model are shown in **Table 3**. The indirect effects collectively accounted for 43.7% of the total effect. Two significant indirect pathways were identified: childhood maltreatment → PB → suicidal ideation and childhood maltreatment → DSO → PB → suicidal ideation. A pairwise comparison of the indirect effects further indicated that the pathway from childhood maltreatment through DSO and PB to suicidal ideation was significantly stronger than the corresponding pathway through PTS and PB (difference in *β* = .006, 95% CI [.002,.013]).

**Figure 1:**
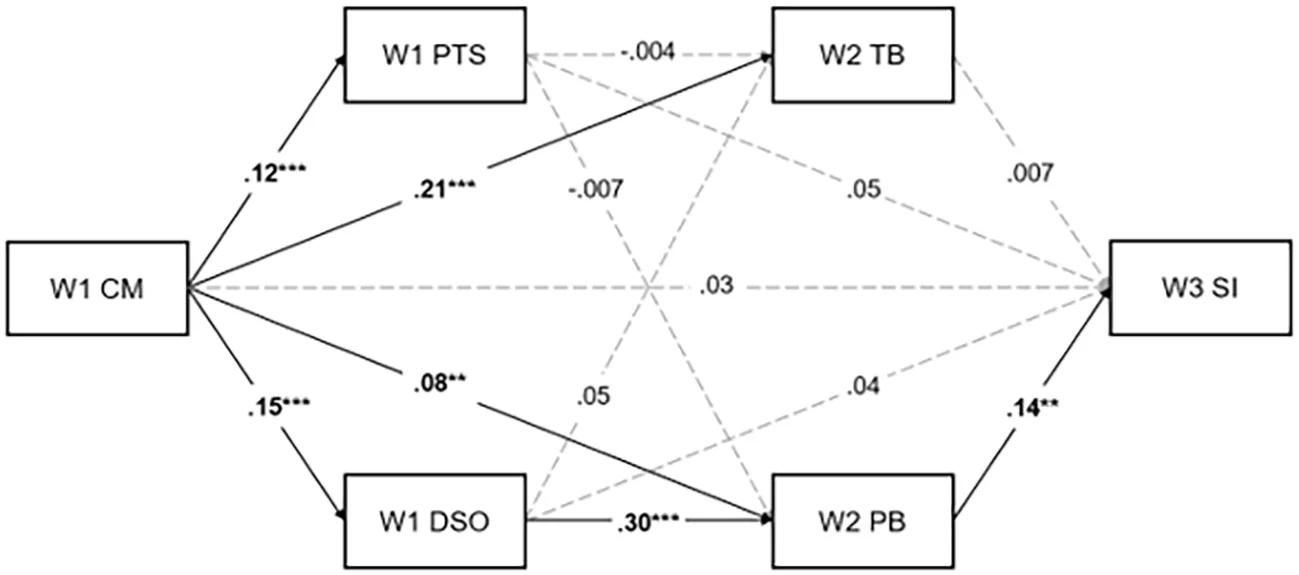
The model of CM, PTS/DSO, TB/PB, and SI (Model 1). W1, Wave 1; W2, Wave 2; W3, Wave 3; CM, Childhood maltreatment; PTS, Posttraumatic stress; DSO, Disturbances in self-organization; TB, Thwarted belongingness; PB, Perceived burdensomeness; SI, Suicidal ideation. **p < .01, and ***p < .001. Solid black lines represent paths with significant coefficients, and dashed gray lines represent paths with insignificant coefficients.

**Table 3:** Standardized indirect path coefficients and bias-corrected bootstrapping test.

Model pathways	*β*	*SE*	95% CI for *β*
Model 1			
CM → PTS → SI	.006	.005	[-.001, .018]
CM → DSO → SI	.006	.006	[-.004, .019]
CM → TB → SI	.001	.006	[-.012, .013]
CM → PB → SI	.012^**^	.006	**[.003, .026]**
CM → PTS → TB → SI	.000	.000	[.000, .000]
CM → PTS → PB → SI	.000	.001	[-.001, .001]
CM → DSO → TB → SI	.000	.000	[.000, .001]
CM → DSO → PB → SI	.006^**^	.003	**[.002, .013]**
Model 2			
CM → AD → SI	.003	.005	[-.005, .014]
CM → NSC → SI	.002	.006	[-.009, .016]
CM → ID → SI	.005	.008	[-.010, .021]
CM → PB → SI	.011^**^	.006	**[.003**, **.026]**
CM → AD → PB → SI	.000	.001	[.000, .002]
CM → NSC → PB → SI	.005^**^	.002	**[.002, .011]**
CM → ID → PB → SI	.001	.001	[.000, .004]

CM, Childhood maltreatment, PTS, Posttraumatic stress, DSO, Disturbances in self-organization, TB, Thwarted belongingness, PB, Perceived burdensomeness, SI, Suicidal ideation, AD, Affect dysregulation, NSC, Negative self-concept, ID, Interpersonal disturbances. ^**^p < .01.Bold indicates that confidence interval does not include zero.

Since PTS and TB did not play significant roles in the model, they were removed, and the final model focused exclusively on the three DSO dimensions. The model fit was acceptable: χ^2^/*df* = 2.986, CFI = .999, TLI = .961, RMSEA [90% CI] = .037 [.000,.0574]. Childhood maltreatment was significantly associated with all three dimensions of DSO (see **Figure 2**). However, only negative self-concept was positively associated with PB, which in turn was prospectively associated with suicidal ideation. The specific indirect effects are presented in **Table 3**. In addition, the full model including all three PTS dimensions and TB can be found in the **Supplementary Materials**.

**Figure 2:**
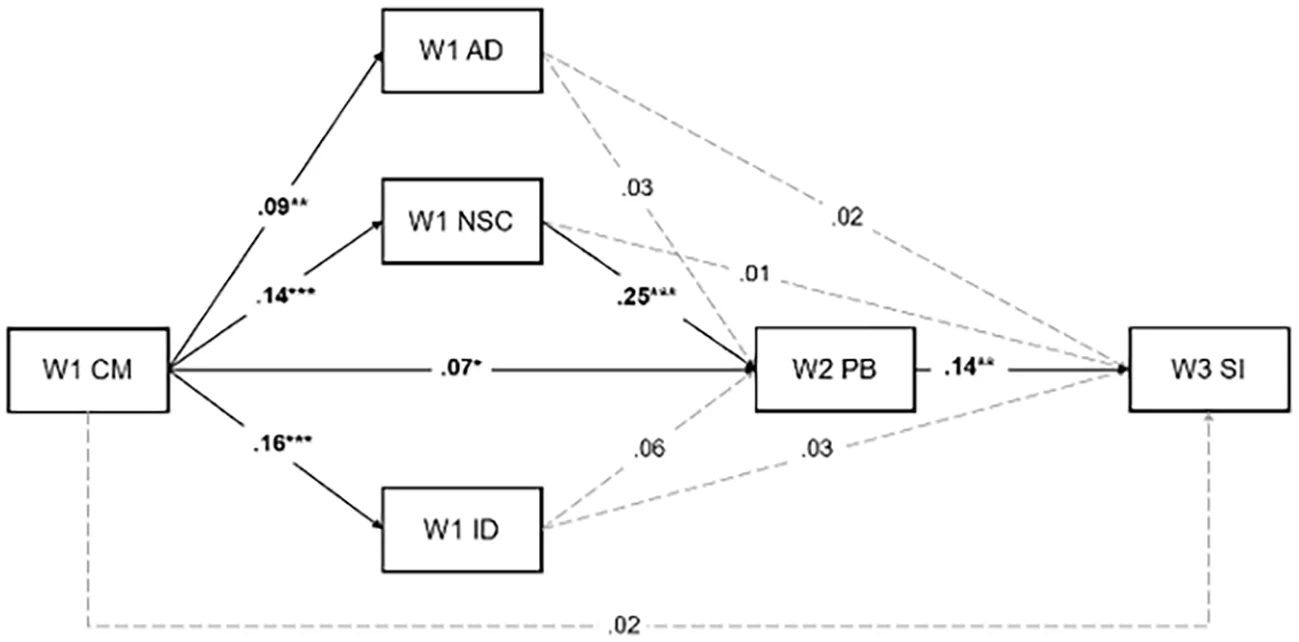
The model of CM, three dimensions of DSO, PB, and SI (Model 2). CM, Childhood maltreatment; PB, Perceived burdensomeness; SI, Suicidal ideation; AD, Affect dysregulation; NSC, Negative self-concept; ID, Interpersonal disturbances. *p < .05, **p < .01, and ***p < .001. Solid black lines represent paths with significant coefficients, and dashed gray lines represent paths with insignificant coefficients.

### 3.3 Sensitivity analysis

Because sex was significantly correlated with childhood maltreatment and PTS in the correlational analyses, we conducted sex-stratified models as sensitivity analyses to explore whether the pathways differed across groups. Detailed results are reported in the **Supplementary Materials** (**Supplementary Figures S2**, **S3**, **Supplementary Table S2**). Among male participants, childhood maltreatment was associated with suicidal ideation primarily through PTS (β = .013, 95% CI [.001,.39]), whereas pathways involving DSO were not statistically significant. Among female participants, the pattern was consistent with that observed in the full sample, with DSO and PB emerging as the primary pathway (β = .010, 95% CI [.004,.023]). These findings suggest that the relative contributions of PTS and DSO may vary by sex.

## 4 Discussion

This study is grounded in the developmental model of suicide risk and the interpersonal theory of suicide, and examines the mechanisms through which childhood maltreatment is associated with suicidal ideation among youth. The results indicate that PB, rather than TB, functions as the primary proximal risk factor for suicidal ideation. Furthermore, within CPTSD, disturbances in self-organization (DSO), rather than posttraumatic stress (PTS), constitute a key pathway linking childhood maltreatment to PB. Specifically, an indirect association was observed between childhood maltreatment and PB through the negative self-concept dimension of DSO, with PB subsequently showing a positive association with suicidal ideation.

### 4.1 The mediating role of CPTSD and interpersonal needs

The findings revealed that PB, rather than TB, mediated the association between childhood maltreatment and suicidal ideation. This result is consistent with prior studies (24, 40), supporting the notion that PB may be a stronger predictor of suicidal ideation than TB (41, 42). For youth with childhood maltreatment, both burdensomeness and loneliness may be elevated. From a theoretical perspective, PB primarily involves negative evaluations of one’s social worth, whereas TB reflects a lack of perceived social connectedness (21). The former is more directly linked to an active desire for death (41). Although TB is also important, it may require the presence of PB, or operate through PB, to indirectly exacerbate suicidal ideation (43).

Notably, the two dimensions of CPTSD exhibited distinct effects. DSO, rather than PTS, linked childhood maltreatment to PB, which was subsequently associated with suicidal ideation. This finding suggests that focusing solely on classical PTSD symptoms may be insufficient to explain the psychological pathways underlying suicide risk among youth with childhood trauma. Prior research indicates that chronic and prolonged trauma, such as childhood maltreatment, not only leads to typical trauma-related symptoms (e.g., rumination and hypervigilance) (44) but also gives rise to disturbances in self-organization, including emotion dysregulation (45), a negative self-concept (46), and avoidance of intimate relationships (47). DSO primarily reflects negative evaluations of the self and others, which are more likely to serve as the source of PB than deficits in stress reactivity. Therefore, for individuals exposed to childhood maltreatment, considering DSO rather than focusing exclusively on PTS may be more critical for understanding the emergence of suicidal ideation.

### 4.2 The different roles of dimensions of DSO

Further analyses indicated that among the three DSO symptoms, negative self-concept primarily served as the mediating factor. Specifically, childhood maltreatment showed an indirect association with PB through negative self-concept within DSO, and PB was subsequently positively associated with suicidal ideation. According to the memory and identity theory of CPTSD (10), an identity centered on worthlessness and inferiority constitutes a core feature of negative self-concept symptoms. Negative self-concept is highly internalized and self-depreciation, directly shaping individuals’ judgments of their social worth and existential significance, which constitute the core of PB (35). When individuals chronically construe themselves as “worthless,” “unworthy of care,” or “a burden to others,” they may be more likely to perceive themselves as burdens to others and society, which is associated with a greater likelihood of suicidal ideation. By contrast, emotion dysregulation primarily reflects difficulties in the intensity and stability of emotional responses, whereas interpersonal disturbances are more indicative of avoidance of intimacy or relational insecurity; although both are closely associated with functional impairment and psychological distress, they do not directly engage core evaluations of self-worth and therefore show relatively limited predictive power for PB.

### 4.3 Sex differences

The sensitivity analyses suggested different pathways across sex-stratified models. Among males, childhood maltreatment was primarily associated with suicidal ideation through PTS, whereas among females, DSO appeared to play a more prominent role. These findings may indicate heterogeneous pathways to suicidal ideation following childhood trauma across sex. Previous research suggests that males may show more prominent PTS responses, with symptoms such as nightmares serving as central features (48), whereas females may be more prone to negative self-concept and interpersonal anxiety, reflected in higher levels of DSO (49, 50). This pattern may suggest that suicidal ideation following childhood trauma is more closely linked to psychopathology responses among males, but to internalizing difficulties among females.

### 4.4 Limitations and implications

Several limitations should be acknowledged. To begin with, the study relied exclusively on self-report instruments, which may introduce recall and reporting bias. Incorporating interview-based assessments and data from multiple sources in future work would likely strengthen measurement precision. In addition, the sample was drawn from a single Chinese college, which may limit the external validity of the findings to populations with different educational backgrounds. Another limitation is that the longitudinal analytic sample was restricted to participants who met the prespecified childhood maltreatment cutoff. Expanding future samples to include more diverse and heterogeneous populations would improve generalizability. Moreover, other mental health symptoms (e.g., depression and hopelessness) and impulsivity may also serve as potential pathways linking childhood maltreatment to suicide risk, particularly among youth with a history of self-injury (51). Future research should control for a broader range of mental health symptoms and trait-like vulnerabilities. In addition, broader interpersonal adversities, such as cyberbullying victimization, have been associated with suicidal ideation among college students (52); future studies should therefore incorporate additional risk and protective factors to better capture the heterogeneity of suicide risk among youth with childhood maltreatment. Another concern is that the relatively low mean score of suicidal ideation may indicate a floor effect and non-normal distribution, which should be considered when interpreting the findings. Future studies should consider robust estimation or alternative modeling approaches for highly skewed suicidal ideation outcomes. Finally, we have clarified that the observed temporal ordering should not be interpreted as evidence of causality. Future research should employ designs with repeated assessments of all focal constructs across multiple waves, control for baseline levels where appropriate, and preferably include more assessment occasions to more rigorously examine within-person longitudinal associations and strengthen causal inference.

Nevertheless, this study provides several important theoretical and practical implications. Theoretically, the findings extend the developmental model of suicide risk by demonstrating the importance of incorporating specific trauma-related symptoms. This contributes to a more nuanced understanding of the developmental pathways to suicidality among youth exposed to childhood maltreatment. In addition, this study differentiated the roles of distinct CPTSD dimensions, highlighting that DSO symptoms exert a mediating effect on suicidality beyond that of PTS symptoms. This finding further underscores the necessity of conceptualizing CPTSD as a distinct diagnostic entity. From a clinical perspective, interventions targeting negative self-concept may reduce PB and thereby help prevent suicidality among youth. Compassion-focused therapy and acceptance and commitment therapy may be particularly beneficial for alleviating maladaptive self-cognitions. Moreover, the sex-stratified findings suggest that interventions targeting PTS may be more relevant for males, whereas greater emphasis on DSO-related difficulties, particularly negative self-concept, may be warranted for females.

## 5 Conclusion

This study found that childhood maltreatment was associated with suicidal ideation among youth through CPTSD symptoms and PB. Within CPTSD, DSO, rather than PTS, primarily accounted for this effect. Within DSO, negative self-concept, rather than emotion dysregulation or interpersonal disturbances, played a central role. These findings highlight the critical role of DSO in the development of suicidal ideation among youth with childhood trauma and underscore the importance of targeting negative self-concept in interventions.

## Data Availability

The original contributions presented in the study are included in the article/**Supplementary Materials**. Further inquiries can be directed to the corresponding author.
