# Supplementary material for: Longitudinal associations between childhood maltreatment and suicidal ideation in youth: the roles of complex posttraumatic stress disorder symptoms and interpersonal factors
Source: Front Psychiatry. 2026 Oct 14;17:1976531. doi: 10.3389/fpsyt.2026.1976531 (PMC13713517; doi:10.3389/fpsyt.2026.1976531)
Supplement: Supplementary file 1 [file SupplementaryFile1.docx]

**Figure S1**

*The model of CM, three dimensions of DSO/PTS, TB, PB, and SI*

*Note.* W1 = Wave 1, W2 = Wave 2, W3 = Wave 3, CM = Childhood maltreatment, RE = Re-experiencing, AV = Avoidance, SOT = Sence of threat, AD = Affect dysregulation, NSC = Negative self-concept, ID = Interpersonal disturbances, TB = Thwarted belongingness, PB = Perceived burdensomeness, SI = Suicidal ideation. ^**^*p* < .01, and ^***^*p* < .001. Solid black lines represent paths with significant coefficients, and dashed gray lines represent paths with insignificant coefficients.

**Figure S2**

*The model of CM, three dimensions of DSO/PTS, TB, PB, and SI in the male group*

*Note.* W1 = Wave 1, W2 = Wave 2, W3 = Wave 3, CM = Childhood maltreatment, RE = Re-experiencing, AV = Avoidance, SOT = Sence of threat, AD = Affect dysregulation, NSC = Negative self-concept, ID = Interpersonal disturbances, TB = Thwarted belongingness, PB = Perceived burdensomeness, SI = Suicidal ideation. †*p* < .08, ^**^*p* < .01. Solid black lines represent paths with significant coefficients, and dashed gray lines represent paths with insignificant coefficients.

**Figure S3**

*The model of CM, three dimensions of DSO/PTS, TB, PB, and SI in the female group*

*Note.* W1 = Wave 1, W2 = Wave 2, W3 = Wave 3, CM = Childhood maltreatment, RE = Re-experiencing, AV = Avoidance, SOT = Sence of threat, AD = Affect dysregulation, NSC = Negative self-concept, ID = Interpersonal disturbances, TB = Thwarted belongingness, PB = Perceived burdensomeness, SI = Suicidal ideation. ^*^*p* < .05, ^**^*p* < .01. Solid black lines represent paths with significant coefficients, and dashed gray lines represent paths with insignificant coefficients.

**Table S1**

*Standardized indirect path coefficients and bias-corrected bootstrapping test*

| Model pathways | *β* | *SE* | 95% CI for *β* |
| --- | --- | --- | --- |
| CM → RE → TB → SI | .000 | .000 | [.000, .000] |
| CM → RE → PB → SI | .000 | .001 | [-.001, .002] |
| CM → AV → TB → SI | .000 | .000 | [.000, .000] |
| CM → AV → PB → SI | .000 | .001 | [-.001, .002] |
| CM → SOT → TB → SI | .000 | .000 | [.000, .001] |
| CM → SOT → PB → SI | .000 | .001 | [-.002, .002] |
| CM → AD → TB → SI | .000 | .001 | [-.001, .001] |
| CM → AD → PB → SI | .000 | .001 | [-.001, .002] |
| CM → NSC → TB → SI | .000 | .000 | [.000, .001] |
| CM → NSC → PB → SI | .005^***^ | .002 | **[.002, .011]** |
| CM → ID → TB → SI | .000 | .001 | [-.002, .002] |
| CM → ID → PB → SI | .001 | .001 | [-.001, .004] |

*Note.* CM = Childhood maltreatment, RE = Re-experiencing, AV = Avoidance, SOT = Sence of threat, AD = Affect dysregulation, NSC = Negative self-concept, ID = Interpersonal disturbances, TB = Thwarted belongingness, PB = Perceived burdensomeness, SI = Suicidal ideation. *^***^p* < .001.

**Table S2**

*Standardized indirect path coefficients and bias-corrected bootstrapping test in males and females*

| Model pathways | *β* | *SE* | 95% CI for *β* |
| --- | --- | --- | --- |
| Male |  |  |  |
| CM → PTS → SI | .013 | .009 | **[.001, .036]** |
| CM → DSO → SI | .002 | .009 | [-.013, .025] |
| CM → TB → SI | .001 | .009 | [-.018, .018] |
| CM → PB → SI | .009 | .007 | [-.001, .028] |
| CM → PTS → TB → SI | .000 | .000 | [-.001, .001] |
| CM → PTS → PB → SI | .000 | .001 | [-.003, .001] |
| CM → DSO → TB → SI | .000 | .000 | [-.001, .001] |
| CM → DSO → PB → SI | .004 | .003 | [.000, .013] |
| Female |  |  |  |
| CM → PTS → SI | .001 | .006 | [-.009, .017] |
| CM → DSO → SI | .010 | .007 | [-.001, .029] |
| CM → TB → SI | .002 | .010 | [-.016, .022] |
| CM → PB → SI | .013 | .010 | [.000, .040] |
| CM → PTS → TB → SI | .000 | .000 | [.000, .000] |
| CM → PTS → PB → SI | .000 | .001 | [-.002, .003] |
| CM → DSO → TB → SI | .000 | .000 | [-.001, .002] |
| CM → DSO → PB → SI | .010^***^ | .004 | **[.004, .022]** |

*Note.* CM = Childhood maltreatment, PTS = Posttraumatic stress, DSO = Disturbances in self-organization, TB = Thwarted belongingness, PB = Perceived burdensomeness, SI = Suicidal ideation, AD = Affect dysregulation, NSC = Negative self-concept, ID = Interpersonal disturbances. *^**^p* < .01.
